# Transitioning couple’s voluntary HIV counseling and testing (CVCT) from stand-alone weekend services into routine antenatal and VCT services in government clinics in Zambia’s two largest cities

**DOI:** 10.1371/journal.pone.0185142

**Published:** 2017-10-16

**Authors:** Mubiana Inambao, William Kilembe, Lauren A. Canary, Nancy L. Czaicki, Matilda Kakungu-Simpungwe, Roy Chavuma, Kristin M. Wall, Amanda Tichacek, Julie Pulerwitz, Ibou Thior, Elwyn Chomba, Susan A. Allen

**Affiliations:** 1 Rwanda Zambia HIV Research Group, Department of Pathology & Laboratory Medicine, School of Medicine and Hubert Department of Global Health, Rollins School of Public Health, Emory University, Atlanta, GA, United States of America; 2 Zambia Emory HIV Research Project (ZEHRP), Ndola, Zambia; 3 Zambia Emory HIV Research Project (ZEHRP), Lusaka, Zambia; 4 District Health Management team (DHMT), Ministry of Community Development, Maternal and Child Health (MCDMCH), Lusaka, Zambia; 5 Department of Epidemiology, Rollins School of Public Health, Emory University, Atlanta, Georgia, United States of America; 6 PATH, Washington, DC, United States of America; 7 Ministry of Community Development, Maternal and Child Health (MCDMCH), Lusaka, Zambia; Yale University Yale School of Public Health, UNITED STATES

## Abstract

**Introduction:**

Most HIV infections in Africa are acquired by married/cohabiting adults and WHO recommends couple’s voluntary HIV counseling and testing (CVCT) for prevention. The handover from NGO-sponsored weekend CVCT to government-sponsored services in routine weekday antenatal care (ANC) and individual voluntary testing and counseling (VCT) services in Zambia’s two largest cities from 2009–2015 is described.

**Methods:**

Government clinic counselors were trained to provide CVCT, and along with community health workers they promoted CVCT services in their clinic and surrounding areas. When client volume exceeded the capacity of on-duty staff in ANC and VCT, non-governmental organization (NGO) subsidies were offered for overtime pay.

**Results:**

Implementation of routine CVCT services varied greatly by clinic and city. The 12 highest volume clinics were examined further, while 13 clinics had CVCT numbers that were too low to warrant further investigation. In Lusaka, the proportion of pregnant women whose partners were tested rose from 2.6% in 2009 to a peak of 26.2% in 2012 and 24.8% in 2015. Corresponding reports in Ndola were 2.0% in 2009, 17.0% in 2012 and 14.5% in 2015. Obstacles to CVCT included: limited space and staffing, competing priorities, record keeping not adapted for couples, and few resources for promotion and increasing male involvement. Conflicting training models for ‘partner testing’ with men and women separately vs. CVCT with joint post-test counseling led to confusion in reporting to district health authorities.

**Discussion:**

A focused and sustained effort will be required to reach a meaningful number of couples with CVCT to prevent heterosexual and perinatal HIV transmission. Establishing targets and timelines, funding for dedicated and appropriately trained staff, adoption of standardized data recording instruments with couple-level indicators, and expansion of community and clinic-based promotions using proven models are recommended.

## Introduction

Sub-Saharan Africa is home to over 68% of people living with HIV [1]. Despite many efforts to halt the epidemic, the virus continues to spread, especially in resource-limited communities. In Zambia, with a population of over 16 million people in 2015 [2] approximately 60% of adults ages 15–49 are married and one in eight adults has HIV [3]. Additionally, in the capital city of Lusaka, approximately 23% of cohabiting couples are discordant (one partner is positive and the other is negative) [4] and corresponding data from Ndola show 16% of couples discordant [5, 6]. However, in 2011 only 16% of individuals ages 15–49 knew their HIV status and an estimated 10%-20% of Zambian couples are unknowingly living with an opposite-serostatus partner [7].

Uninfected (HIV-) pregnant women with a positive (HIV+) male partner put themselves and their child at risk for HIV infection. The HIV prevalence rate of 11.6% among pregnant women in in Zambia and most pregnant women are married or cohabiting [8], thus integrating CVCT into antenatal care is a valuable way to prevent horizontal (prong 1 of PMTCT) [9] and vertical transmission of HIV.

Supporting this initiative, current prevention of mother-to-child transmission (PMTCT) guidelines in Zambian antenatal clinics (ANC) specify testing the male partner in addition to the mother[9]. CVCT is a cost-effective way to accomplish this while ensuring mutual disclosure [10–12] and could prevent an estimated half of new infections in Zambian couples [13]. In addition, an external review of Zambia’s PMTCT policies deemed male involvement in ANC programs an essential component of PMTCT and identified CVCT as a best practice for increasing it [14].

The Zambia-Emory HIV Research Project (ZEHRP), in collaboration with the Ministry of Health (MoH) and the Ministry of Community Development, Maternal & Child Health (MCDMCH) has been implementing CVCT in the Republic of Zambia since 1994 in both stand-alone and government clinic settings [15–22]. In 2008, the MoH endorsed CVCT and requested that ANC departments include male partners in HIV testing. In this study we examine progress with partners testing in ANC since that time as reflected in data reported to government. Data extraction from government logbooks is used to assess the degree to which couples were jointly post-test counseled separately (partner testing) or together (CVCT) in both ANC and voluntary HIV counseling and testing (VCT) services. Through observations and discussions with clinic staff, we also explore challenges in integrating CVCT into these routine services and propose solutions.

## Methods

### Clinics, training and timeframes

Beginning in 2008, ZEHRP trained government clinic counselors and health promoters in 25 clinics in Lusaka (N = 12) and Ndola (N = 13) to promote and provide CVCT and paid overtime to off-duty clinic staff to offer these services on weekends when the clinics were not congested. In addition to periodic radio programs, promotions were carried out in the community by District Clinic Promoters (DCP) affiliated with the clinics, and by Influence Network agents (INA) affiliated with ZEHRP [5, 21, 22]. In addition, clinic nurses and counselors promoted CVCT among ANC and VCT clients. The same government nurses and counselors that provided CVCT on the weekends also worked in their respective ANC and VCT clinic departments during the week and thus had the skills to provide CVCT during their normal working hours. To support integration of CVCT into routine ANC and VCT in high volume clinics, ZEHRP offered to subsidize an additional off-duty clinic counselor to increase capacity to provide CVCT on weekdays if clinic staff on duty were receiving > = 5 couples/day and could not cope with the workload.

### Data collection

Lusaka and Ndola clinics reported ANC partner testing to their District Health Management Teams (DHMT) through the Health Medical Information System (HMIS, aggregated data unlinked from identifiers). HMIS data for partner testing in ANC was included for the years that it was available (2009–2015) to show citywide trends of partner testing. HMIS data included men who were tested separately from their pregnant wives as well as those who were jointly post-test counseled with facilitated disclosure.

To assess the accuracy of HMIS data regarding partner testing in ANC, determine what proportion of couples were receiving CVCT with joint post-test counseling, and evaluate CVCT in VCT services, data extraction from government issued VCT and either ANC and/or PMTCT logbooks was conducted in 25 clinics. For comparison of volume across years, data for the same months, from February to May, of each year was manually extracted from clinic logbooks annually in 2011–2013 in the months immediately following the measurement period. Notes were taken from discussions with clinic staff and observations of partner testing and CVCT services in parallel with data abstraction.

For 2011–2013 where both data sources were present, HMIS data was compared to data extracted for this study to discern whether and when couples were receiving joint counseling versus separate partner testing, identify discrepancies and variations in source documents and tallying procedures, and inform strategies for improved reporting. CVCT provided by ‘on duty’ clinic staff (sponsored by government) and ‘off duty’ counselors (paid by ZEHRP) were counted separately to assess how well each clinic was moving toward independence from NGO resources.

In both cities, clinic staff was asked about procedures used when couples requested HIV testing and how data was recorded. Such practices were also informally observed to gain insight into how CVCT was being incorporated into routine services. The logbooks used were identified by clinic staff who explained how data was recorded and tallied, walked the researchers through the process, and remained available for questions during data extraction. Emory School of Public Health students worked alongside medical students from the University of Zambia under the supervision of the DHMT Directors in each city and with the assistance of clinic staff and the sister in charge. Though personal identifying information was used to link men and women as couples, no patient identifiers were collected or recorded by researchers. The only information recorded was the total number of couples tested by month and department.

For our purposes, CVCT was defined as a man and woman that received HIV counseling and testing on the same day and received their results and post-test counseling together. It is important to note that this number of couples tested is not equivalent to ANC partners tested as reported in the HMIS DHMT reports. The latter also includes women and male partners who were tested separately, often on different days, without counselor-facilitated disclosure and joint post-test counseling. Since the effectiveness of CVCT rests on counseling and disclosing results together, data extraction from clinic logbooks focused only on CVCT and not separate partner testing.

Staff assistance during data collection ensured consistent data extraction strategies across departments and clinics. When couples were unable to be verified either by staff or additional documentation as having received post-test counseling and test results together, they were not counted as a CVCT couple.

Regulatory approvals: The Emory University Institutional Review Board determined that ethical review was not required because the study did not meet the federal definition of research with human subjects or clinical investigation. The study was also reviewed by the Ministry of Community Development and Maternal and Child Health (co-author Chomba) and the Directors of the District Health Management Teams (DHMT) in Ndola (co-author Simpungwe) and Lusaka (co-author Chavuma) districts of Zambia. Permissions were obtained from the sisters in charge at each clinic to work alongside staff to extract data and observe the flow of couple and partner testing activities. Individual CVCT sessions were not observed.

### Data analysis

HMIS data are presented by city and by year and includes the number and proportion of new ANC clients whose partners were tested from 2009–2015. Monthly averages of CVCT clients were calculated per clinic, per department (ANC, VCT) and per sponsor (government, ZEHRP) from data extracted from 2011–2013. For the subset of clinics with adequate numbers of CVCT clients, results were graphed by year and by department to illustrate trends and variations between clinics.

## Results

### Lusaka

**Fig 1:**
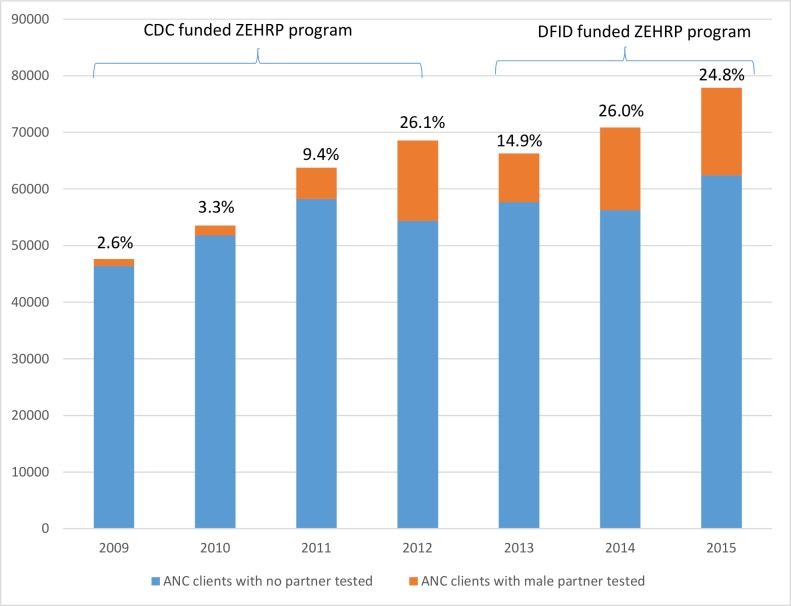
Number and percent first time ANC clients and partners tested during 2009–2015, from HMIS reports to DHMT in Lusaka. Blue represents women whose partners were not tested, orange represents women whose partners were tested. Percentage of ANC clients with male partner tested is shown above each bar.

Aggregate partner testing data from ANC as reported to the Lusaka DHMT through the HMIS system are shown in the aggregate in Fig 1 and confirm a steady increase from 2.6% of antenatal clients’ male partners tested in 2009 to 26.1% tested in 2012. There was a drop to 14.9% in 2013 which could be attributed in part to transition of ZEHRP funding from CDC (all couples) to DFID funding (non-pregnant couples only). Encouragingly, the percentage rebounded increased again to 26% in 2014 and 24.8% in 2015. The raw numbers of pregnant women in Lusaka increased steadily from over 45,000 in 2009 to close to 80,000 in 2015 reflecting rapid growth in Zambia’s capital and largest city.

**Fig 2:**
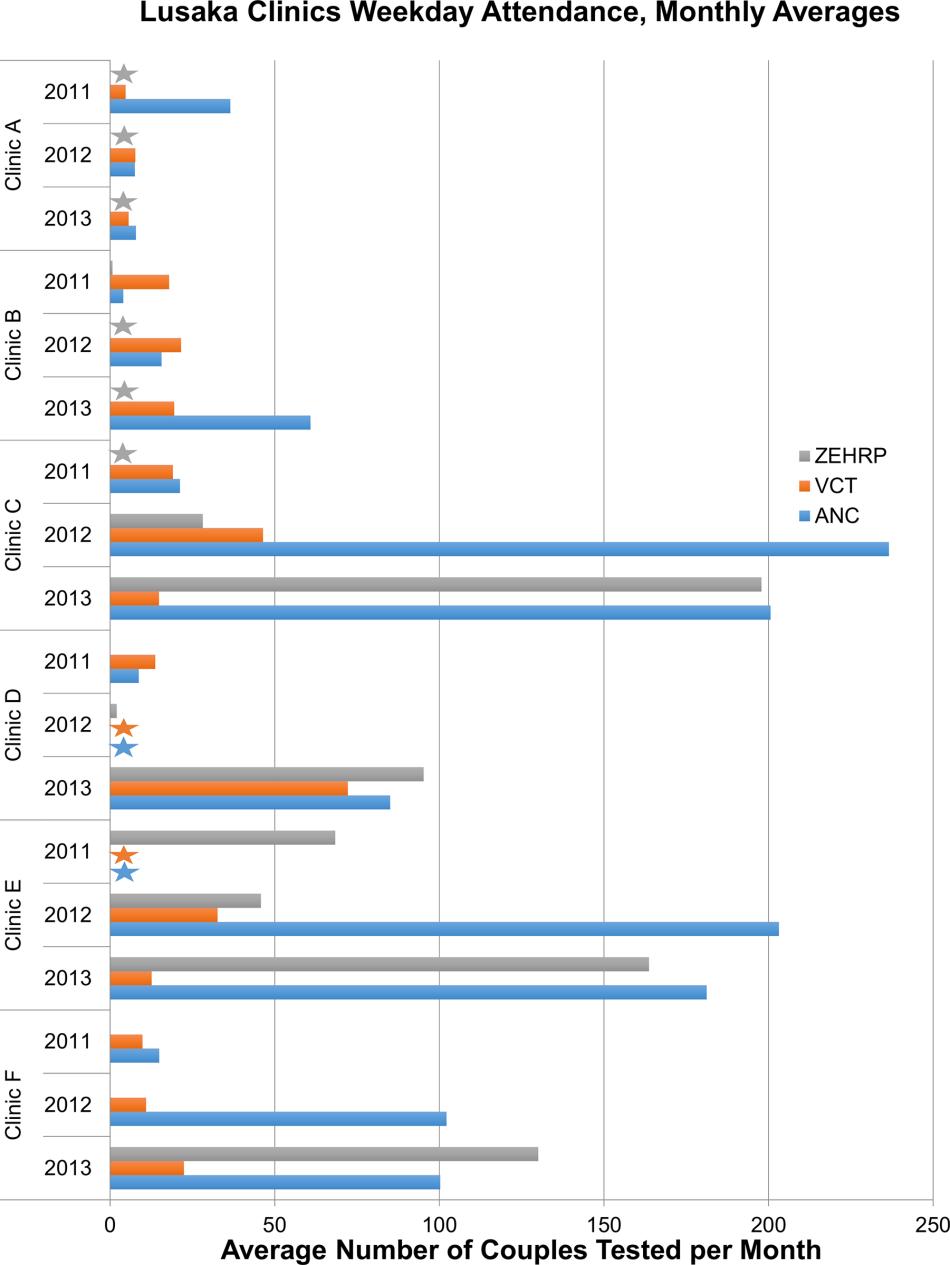
Lusaka: Average number of couples per month that received weekday couples voluntary counseling and testing in clinics with a monthly average of ≥40 couples in at least one year. Blue bar represents CVCT provided by on-duty government counselors in the ANC clinics; orange bars represent CVCT provided by on duty government counselors in the VCT department; grey bar represents weekday CVCT provided by a ZEHRP-sponsored counselor. Grey stars indicate clinic-years when no ZEHRP-sponsored staff provided CVCT services. Blue and orange stars indicate clinics in which logbooks for data extraction were not available for that year.

Data extraction in 12 clinics showed only six with a monthly average of ≥40 couples in at least one year: these de-identified and included in Fig 2. Two clinics lacked ‘on duty’ ANC and VCT data for one year, but are included because the available data show useful trends. Monthly averages for the remaining six clinics ranged from 3–18 couples per month (data not shown), indicating poor achievement with integrating CVCT. These clinics are not examined further.

Five of the six Lusaka clinics showed an increase in testing during routine government-funded weekday services from 2011 to 2013. Four clinics that were able to reach a minimum of 5 couples/day in ANC departments made use of the additional off duty CVCT counselor sponsored by ZEHRP (grey bar), while other clinics either did not reach that benchmark or were able to manage the volume without assistance (orange and blue bars).

Though broadly ANC CVCT volume was higher than that found in VCT, there was considerable variation between clinics. Clinic A was the only high-performing clinic in 2011 that experienced a decrease in subsequent years, dropping from 41 couples/month in 2011 to 15/month in later years. Clinic B saw an increase from 23/month in 2011 to 80/month in 2013 with steady levels in VCT and increases in ANC. Clinic C had the highest number of couples served by government-sponsored staff in 2012. Though the overall number increased in 2013, half of ANC couples were served by a ZEHRP-sponsored off-duty nurse, indicating that government staff were not able to keep up with increasing demand. Similar patterns were evident in the next highest performing clinics, E and F, which experienced dramatic increases in the number of couples tested paralleled by increased support from ZEHRP. Clinic D was unique in having almost equal numbers in ANC and VCT in 2013, having risen substantially from 2011 to 2013, though 2012 data was not available.

The highest volume clinics were located in neighborhoods that had previously hosted a neighborhood randomized control trial of CVCT promotion (NIMH R0166767; 2003–2007) and/or other ZEHRP-sponsored work involving promotion and provision of CVCT from the late 1990’s onward [4, 15–18, 21, 22].

### Ndola

**Fig 3:**
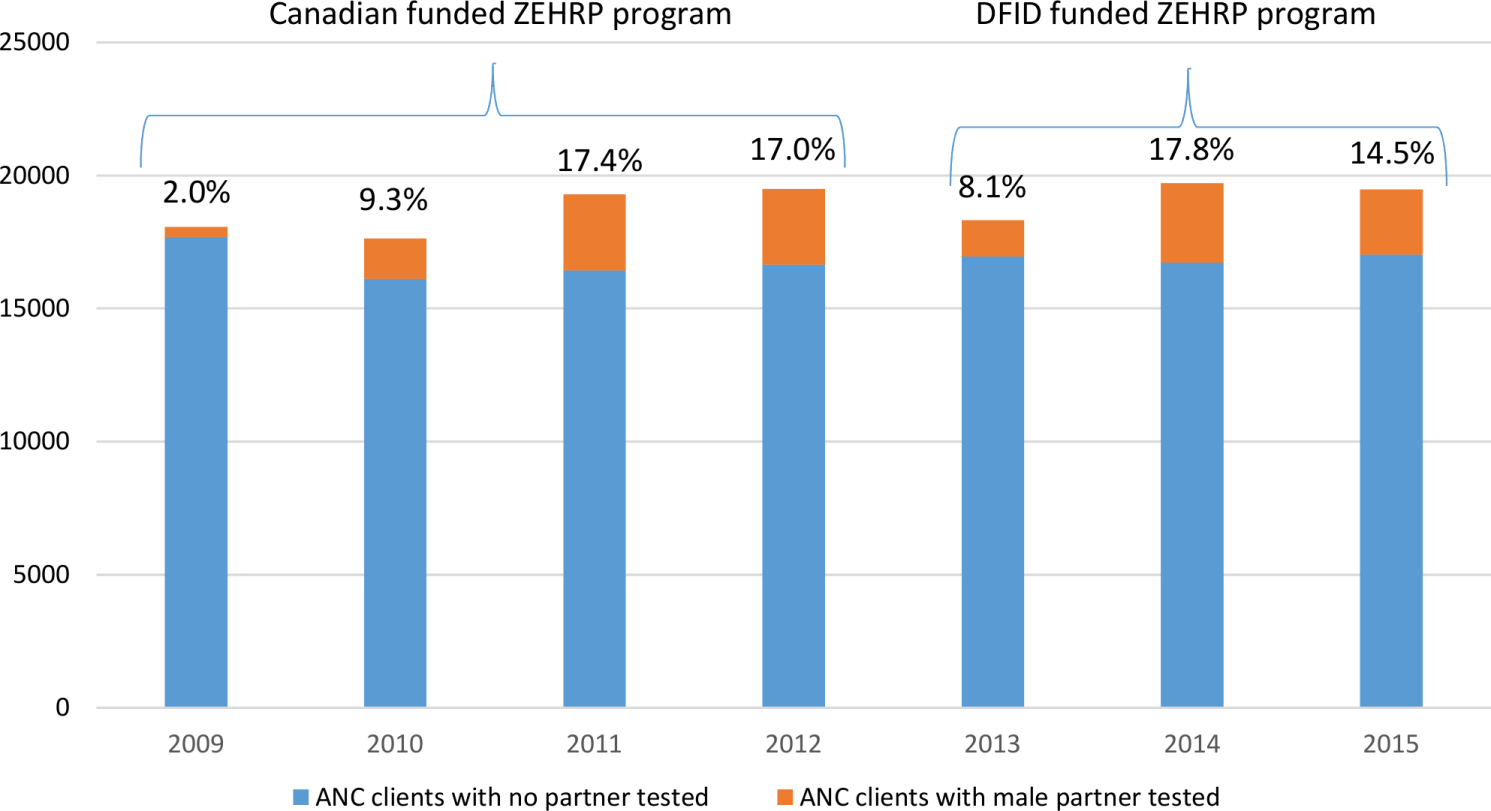
Number and percent of first time ANC clients and partners tested 2009–2015, from HMIS reports to DHMT in Ndola. Blue represents women whose partners were not tested, orange represents women whose partners were tested. Percentage of ANC clients with male partner tested is shown above each bar.

Aggregate partner testing data from ANC as reported to the Ndola DHMT through the HMIS system are shown in the aggregate in Fig 3. In contrast to the dramatically increased numbers of pregnant women observed in Lusaka, ANC intake in Ndola remained level at 17,000–19,000/year over the same time period. As in Lusaka, Ndola showed a steady increase from 2.0% of antenatal clients’ male partners tested in 2009 to 17.4% tested in 2011 and 17.0% 2012. Also as in Lusaka, there was an initial drop to 8.1% in 2013 when Canadian funding (all couples including those in ANC) transitioned to DFID funding (non-pregnant couples only, thus excluding ANC)), but the percentage increased again in 17.8% in 2014 and 14.5% in 2015.

**Fig 4:**
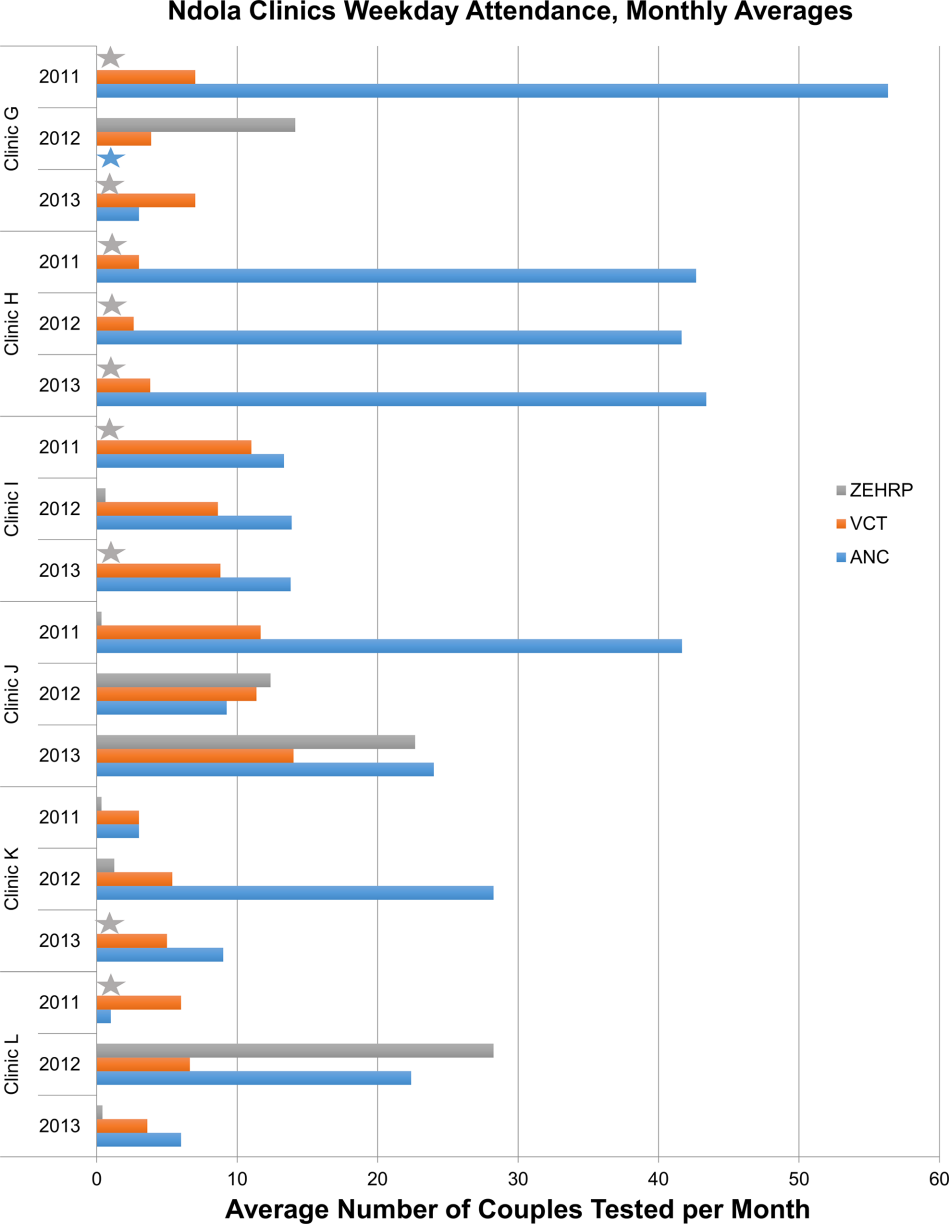
Ndola: Average number of couples per month that received weekday couples voluntary counseling and testing in clinics with at least two years of data. Blue bar represents CVCT provided by on-duty government counselors in the ANC clinics; orange bars represent CVCT provided by on duty government counselors in the VCT department; grey bar represents weekday CVCT provided by a ZEHRP-sponsored counselor. Grey stars indicate clinic-years when no ZEHRP-sponsored staff provided CVCT services. Blue stars indicate clinics in which logbooks for data extraction were not available for that year.

Data extraction in 13 clinics yielded 6 with attendance data showing some success for 2011, 2012 and 2013 (Fig 4**)**. The remaining 7 clinics had small numbers and were not further considered. Only clinic H maintained averages of >40 couples/month in all 3 years. Three clinics (G, J, and L) tested an average of ≥40 couples/month in at least one year; two (Clinic I and K) had 24 and 35/month at their highest point respectively. As in Lusaka, clinics varied with clinic I having comparable numbers in ANC and VCT while other clinics had predominately ANC couples. Four clinics (G,J,K,L) made use of ZEHRP-sponsored overtime staff to help manage demand at some point while the others were able to manage the volume with only government sponsored counselors. Three clinics (G, K, and L) showed declining numbers over time. The highest volume clinics were located in neighborhoods that had hosted ZEHRP-sponsored CVCT promotions [5, 6].

### Challenges to integrating CVCT

Through informal conversations with staff and clinic in-charges and observation of the flow of clients and partners, we identified several obstacles to integrating CVCT into routine practice. Procurement procedures were an obstacle because ANC test kits could only be used for women, thus requiring male partners to be referred to VCT for blood draw and rapid testing. There were also conflicting training programs with ZEHRP stressing that couples should receive results together and another NGO advising separate post-test counseling in view of the separate flow of test kits in ANC and VCT. This led to confusion about what exactly was CVCT and how it should be implemented and reported. Additionally, some clinics had implemented incentives such as jumping the queue if the woman comes with her partner. Others had adapted ZEHRP’s models of clinic and community-based promotions [5, 21, 22] and/or written invitations to women’s partners to encourage them to come to clinic. Despite these efforts, limited space, lack of resources, understaffing, and long wait times (the entire process could take up to 4–5 hours from the time of arrival at the clinic) were often a barrier to implementing CVCT.

Though difficult to measure, leadership and management came up repeatedly as a reason why CVCT services were or were not being well incorporated into routine services. Sisters in charge and staff are routinely rotated between clinics every 2–3 years and clinic leadership is given flexibility and autonomy with management decisions. Lastly, as described above clinics in neighborhoods that had a longer history of collaboration with ZEHRP research and promotional programs had better success with transitioning from NGO to government-sponsored CVCT.

### Challenges in data collection and reporting of CVCT using existing tools

VCT logbooks were used to identify couples tested together in the VCT department, but they varied extensively and were often hand-written rather than printed. CVCT was not a reportable indicator in VCT logbooks and recording was inconsistent. Couples coming to VCT together were sometimes marked with an asterisk or a “C”. Couples could also be identified by finding a man and woman with the same surname listed sequentially.

For ANC couples, women were recorded in the ANC or PMTCT logbook while male partners were recorded in the VCT logbook described above. The ANC logbook included a column for “partner tested” but this was not used consistently. Some counselors used ‘N’ for ‘negative’ and ‘P’ for ‘positive’, while others used ‘N’ for ‘not tested’ and ‘Y’ for ‘tested’. In these situations, N could have been ‘Not tested’ or ‘Negative’. These ANC/PMCT logbooks were the most common source for the tallies reported to the DHMT, and without further analysis it was not possible for staff to distinguish couples counseled separately from those counseled together.

During data extraction, if the ANC “partner tested” column was filled with “N”, “Y”, “P” or another marking, the researchers searched for the male partner in the VCT logbook using names, locator information and dates. There were some discrepancies across and within clinics on this method, with some clinics identifying members of a couple with asterisks in the VCT logs and others not. Not surprisingly, there were many differences in the numbers reported to the DHMT when compared to those extracted from ANC/PMTCT/VCT logbooks. In some cases, DHMT numbers were higher due to the inclusion of partners tested separately, while in others the data extraction yielded higher numbers due to the additional effort made to link women and men across logbooks.

## Discussion

We examined the transition of CVCT from an NGO-funded, stand-alone service offered on weekends in government clinics to a government-funded, integrated service offered on weekdays in routine antenatal and voluntary HIV counseling and testing services in Zambia’s two largest cities. Some clinics were successful in modestly increasing couples’ testing and counseling in weekday services while others were not. The lack of consistency across clinics and over time indicates that clinic and community-level factors play a pivotal role along with national, provincial or district-level trends. We also identified and examined challenges in integrating CVCT into routine services and recording data on these activities. We found that while all clinics had CVCT-trained counselors, none had resources for promotional activities and many did not have sufficient staffing and/or space to host male partners of ANC clients. Procurement procedures restricting ANC test kits to pregnant women (and not their partners), conflicting training programs recommending individual versus joint post-test counseling, and inconsistent adaptation of data recording tools to accommodate couples hampered service delivery and reporting. We describe below successful strategies, discuss challenges and potential solutions, and offer recommendations for both increasing CVCT integration and ensuring a sustainable handoff of best practices from NGOs to government clinics.

Sustained promotions have a strong effect on demand for CVCT [20, 22]. The highest and most sustained CVCT uptake was found in clinics serving communities that had previously hosted NGO-sponsored CVCT services and/or randomized trials of community-based CVCT promotional strategies. For example, Clinic F and Clinic C are the clinics closest to our research operations in Lusaka where we have been promoting and providing CVCT since 1994 [4, 15, 16]. Clinic F was also the site of a pilot study of CVCT for ANC clients in 2001, as was Clinic E [18]. The Clinic E neighborhood also hosted a randomized control trial of CVCT promotional strategies from 2003–2006 [17, 21, 23] Similarly our Ndola research site has promoted and provided CVCT since 2003 [5, 6] and ZEHRP has supported weekend CVCT in Copperbelt clinics in 3 cities including Ndola since 2010.

Written invitations for men to excuse them from work, client incentives such as ‘jumping the queue’ if you bring your partner, and complementary health services (syphilis screening, deworming) were reported by some clinics though the lack of documentation made linking these efforts to service numbers difficult. Other studies have also highlighted the success of such non-coercive strategies in Lusaka and other areas of sub-Saharan Africa [24–26]. For example, while studies in Mozambique have shown that gender ineuqality and stigma are barriers for CVCT uptake by men in ANC [27], this can be overcome by male-to-male community health workers (termed “Male Champions” to create male-friendly norms to engage men in testing with partners in ANC [28]. Similarly, a cluster-randomized trial in Uganda showed that CVCT demand-creation strategies that were couple and male-focused and included promotions by 'expert couples' who had experience with couple’s testing improved uptake of CVCT [29]. These and other approaches for ‘men friendly’ CVCT services should be explored, with consistent funding to support successful promotional techniques and documentation to allow monitoring and evaluation. Care should be taken to avoid coercive strategies that might reduce women’s access to ANC [30] or cause disruption in the home due to lack of information [31].

Even with promotions and sensitization, factors such as stigma, trust and communication within the relationship, inability to be excused from work, and lack of knowledge that one’s HIV results can differ from one’s partner [32–34] [35, 36] likely contribute to low levels of male involvement and should be addressed at the clinic and community-level. Of note, lower-performing clinics in Lusaka and Ndola were excluded from this analysis and may have experienced additional challenges not represented by the clinics cited here.

Counselors from clinics included in this analysis had received ZEHRP CVCT training based on US CDC Guidelines [37] which was uniform across sites, ensuring that the requisite skills were available. However, logistics such as limited space and number of staff contributed to the variability in couples tested across clinics. Space is limited at many clinics with only one waiting room accommodating up to 100 ANC clients on a given day. While government nurses had expertise in CVCT, their other duties often took precedence and they were obliged to rely on support from ZEHRP’s NGO-sponsored staff. This was particularly true in Lusaka as clinics struggled to cope with the burgeoning population. These issues combined with long waiting times limited the implementation of CVCT on a larger scale.

In both Lusaka and Ndola, procedures for procurement of HIV rapid test kits presented a significant obstacle to CVCT. In most clinics, ANC test kit procurement was limited to pregnant women while test kits for male partners were procured through VCT. In order to reconcile procurement and utilization numbers, ANC partners were often required to have the fingerpick done in VCT, and VCT and ANC staff then liaised in order to provide joint post-test counseling to the couple together. Some clinics were able to master these complexities. Others simply referred male partners for individual VCT. Additionally, procurement requests were based on recent consumption and stocks of test kits ran out at times if successful demand creation brought more male partners in to ANC. This highlights the need to coordinate demand creation and supply when expanding CVCT programs.

Additional clinic-level factors such as leadership and management are crucial for successful implementation and surveillance of CVCT as decisions about allocation of staff and space are made at this level. Though this is difficult to measure directly, informal discussions with staff members indicated strong, energetic leadership at clinics such as Clinic C and Clinic H, which saw sustainable increases in couples testing.

Though many ANC clinics maintain similar logbooks, data was often difficult to accurately extract as there was no uniform standard for the recording and reporting of data from couples. Pressure to increase ‘partner testing’ resulted in staff testing women alone in ANC while sending partners separately to VCT. While this may have allowed for larger numbers of partners to be tested, the prevention impact of facilitated disclosure was lost and data was less likely to be recorded in the same logbook. Though initial increases in CVCT between 2009 and 2012 were encouraging (2–3% in 2009 to 17% in Ndola and 26% in Lusaka), these were attributable to success in a few clinics with most clinics unable to incorporate couples in the daily routine. To date, three quarters or more of pregnant women are not tested with partners. This represents a missed opportunity for prevention of new infection in men, women, and newborns and one that will not change without a coordinated effort including targets, timelines, dedicated budgets and standardized indicators.

To help improve logistics, management and data quality, data collection instruments in ART and logbooks for ANC and VCT should be revised to incorporate couple level indicators, and these should be required in reporting to the District, Provincial, and National level. Clinics should set achievable attendance targets and track their progress towards meeting those targets. Additionally, clinics should keep a record of any promotional strategies used so that they may be evaluated in relationship to clinic attendance for couples testing. This would allow successful strategies to be shared and disseminated. Standard procedures should be established per WHO guidelines [38] and CDC training materials, which require joint post-test counseling [37].

## Conclusion

Given WHO Guidelines and endorsement of CVCT by Zambia Ministry of Health and Ministry of Community Development, Maternal and Child Health, we make the following recommendations to address the challenges of integrating CVCT into routine government service: 1) sustained demand creation through evidence-based clinic and community-based promotions; 2) advocacy with clinic management and leadership to optimize available staff and space to accommodate CVCT in ANC, VCT and other services; 3) consistent use of WHO guidelines and CDC training materials endorsing joint post-test counseling with mutual disclosure; 4) HIV test kit procurement procedures and data recording and reporting tools facilitating couple-level reporting; and 5) establishment of targets, indicators, and funded implementation plans to monitor number and percent of pregnant women and other coupled adults receiving CVCT.
